# Identification, Characterization, and X-ray Crystallographic Analysis of a Novel Type of Lectin AJLec from the Sea Anemone *Anthopleura japonica*

**DOI:** 10.1038/s41598-018-29498-0

**Published:** 2018-08-01

**Authors:** Hideaki Unno, Azusa Nakamura, Shingo Mori, Shuichiro Goda, Kenichi Yamaguchi, Keiko Hiemori, Hiroaki Tateno, Tomomitsu Hatakeyama

**Affiliations:** 1 Graduate School of Engineering, Nagasaki University, 1-14 Bunkyo-machi, Nagasaki, 852-8521 Japan; 2 Faculty of Fisheries, Nagasaki University, 1-14 Bunkyo-machi, Nagasaki, 852-8521 Japan; 3 Research Center for Medical Glycosciences, National Institute of Advanced Industrial Science and Technology, Tsukuba, 305-8568 Japan

## Abstract

A novel galactose-specific lectin, AJLec (18.5 kDa), was isolated from the sea anemone, *Anthopleura japonica*. AJLec was characterized using the hemagglutination assay, isothermal titration calorimetry (ITC), and glycoconjugate microarray analysis and we found that AJLec has a specificity for galactose monomers and β-linked terminal galactose residues in complex carbohydrates, but not for N-acetylgalactosamine (GalNAc), which is commonly recognized by galactose-binding lectins. The primary structure of AJLec did not show homology with known lectins, and a crystal structural analysis also revealed a unique homodimeric structure. The crystal structure of AJLec complexed with lactose was solved by measuring the sulfur single-wavelength anomalous diffraction (S-SAD) phasing with an in-house Cu Kα source method. This analysis revealed that the galactose residue in lactose was recognized via its O2, O3, and O4 hydroxyl groups and ring oxygen by calcium coordination and two hydrogen bonds with residues in the carbohydrate-binding site, which demonstrated strict specificity for the β-linked terminal galactose in this lectin.

## Introduction

Lectins are ubiquitously distributed in various tissues and body fluids of living organisms. In particular, lectins play a crucial role in the innate immune system of invertebrates through specific binding to polysaccharide-coated pathogenic bacteria. Lectins are classified into 48 families based on their common structures^1^. Among them, C-type lectins and galectins are widely distributed and known to function in various carbohydrate recognition processes^2^. C-type lectins show a requirement for Ca^2+^ to bind to carbohydrates and have common carbohydrate recognition domains (C-type CRD) that show diverse carbohydrate-binding specificities. Many C-type lectins bear domains in addition to CRDs. Therefore, C-type lectins have 16 subfamilies, which are distinguished by their domain architectures^3^. In each of the subfamilies, the domains cooperate to perform a diverse range of functions, including cell-cell adhesion, immune response to pathogens, and apoptosis^4^. On the other hand, galectins show specific binding toβ-galactosides. Galectins have a unique motif that binds to carbohydrates in CRDs with a jelly-roll conformation. Many galectins have now been identified in animals, each of which is differentially expressed in various tissues. These galectins show a broad range of functions, including cellular adhesion, animal development, immune responses, and apoptosis^5^.

In innate immune systems of invertebrates, lectins are thought to play crucial roles as pattern recognition molecules through specific binding to polysaccharide-coated pathogenic bacteria. Some reports have suggested the presence of marine invertebrate lectins with characteristics that are much different from those of other organisms^6,7^. However, compared to the variety of marine invertebrate species that exist, knowledge about marine invertebrate lectins is very limited, till date.

In the present study, we purified a novel galactose-binding lectin named AJLec from the sea anemone *Anthopleura japonica*. Lectins from sea anemone species have not been identified previously. Characterization of AJLec revealed strict specificity for galactose monomers and β-linked terminal galactoses in complex carbohydrates. Its amino acid sequence does not show any similarity with those of known lectins. Analysis of AJLec complexed with lactose revealed its binding mechanism and a structural basis for its specificity.

## Results

### Purification of AJLec from *A. japonica*

Homogenized extraction form *A. japonica* was used for an affinity chromatography with a lactose-Cellufine column. During the chromatography, a single peak appeared after elution with Tris-buffered saline (TBS) containing 100 mM lactose (Supplementary Fig. S1A). The protein was further purified by gel filtration and dialyzed to remove lactose. SDS-PAGE showed that this lectin was a single polypeptide with a molecular mass of 18 kDa under reducing conditions, whereas a molecular mass of 36 kDa was estimated under non-reducing conditions (Supplementary Fig. S1B). Processing of 300 g of *A. japonica* tissue yielded 10 mg of lectin. This 18-kDa lectin purified from *A. japonica* was named AJLec (*Anthopleura japonica* Lectin).

### Sugar-binding specificity of AJLec

The purified AJLec showed significant hemagglutinating activity with rabbit, horse, bovine, chicken, or sheep erythrocytes (Supplementary Table S1). The greatest hemagglutinating activity was shown with rabbit erythrocytes, with a minimum hemagglutinating concentration of 0.65 μg/mL. Therefore, additional hemagglutination assays to examine the effect of divalent ions and carbohydrate inhibition were performed with concentrations of 2.4–2.6 μg/mL AJLec and rabbit erythrocytes. A hemagglutination assay with a series of concentrations of Mg^2+^ and Mn^2+^ ions and without any cations did not show any hemagglutinating activity, whereas that with Ca^2+^ showed significant activity with a minimum concentration of 0.1 mM (Supplementary Table S1). This indicates that AJLec is a Ca^2+^ ion-dependent lectin. The hemagglutinating activity of AJLec was effectively inhibited only by d-galactose and galactose derivatives, including Me-α-Gal, Me-β-Gal, lactose, lactulose, melibiose, and raffinose (Table 1). However, GalNAc, a derivative of galactose, did not show significant inhibition. Affinity of AJLec for galactose and lactose was measured using isothermal titration calorimetry (ITC) (Table 2 and Supplementary Fig. S2). Galactose and lactose affinities were characterized by the association constants 8.28 ± 1.85 × 10^3^ M^−1^ and 11.3 ± 1.86 × 10^3^ M^−1^, respectively. Although binding stoichiometry was calculated to be 0.482 (galactose) and 0.561 (lactose), these values may be underestimated because of the low association constants of the lectin^8^. In fact, the crystal structure of AJLec/galactose complex revealed that there is one carbohydrate-binding site in each polypeptide chain as described below.

**Table 1:** Inhibition of the hemagglutination activity of AJLec by sugars and glycoproteins.

Sugars	Minimum inhibitory concentration, mM
d-Galactose	0.05
Me-α-Gal	0.8
Me-β-Gal	0.4
*N-*Acetylgalactosamine	25
d-Glucose	>25
Me-α-Glc	>25
d-Mannose	>25
Me-α-Man	>25
l-Fucose	3
L-Rhamnose	3
Lactose (Galβ1-4Glc)	0.1
Lactulose (Galβ1-4Fru)	0.1
Melibiose (Galα1-6Glc)	0.8
Raffinose (Galα1-6βGlc1-2Fru)	0.8
Palatinose(Glcα1-6Fru)	3.1
Maltose (Glcα1-4Glc)	6.3
**Minimum inhibitory concentration, mg/mL**	
Mannan	>2.5
Porcine stomach mucin	>2.5
Fetuin	2.5

**Table 2:** Thermodynamic characteristics of the binding of AJLec to carbohydrates.

Sugar	*n*	*K*
		×*10*^*3*^ *M*^−1^
Galactose	0.482	8.28 ± 1.85
Lactose	0.561	11.3 ± 1.86

Hemagglutinating activity was not inhibited by mucin, whereas weak inhibition was observed with fetuin. Galactose residues are present in the part of carbohydrates modified by mucin^9^ and fetuin^10^. We speculated that the binding ability of AJLec was influenced by the orientation and/or positions of galactose residues in complex carbohydrates based on the results obtained on treatment with mucin and fetuin. For further analysis, a glycoconjugate microarray analysis was performed.

### Glycoconjugate microarray

**Figure 1:**
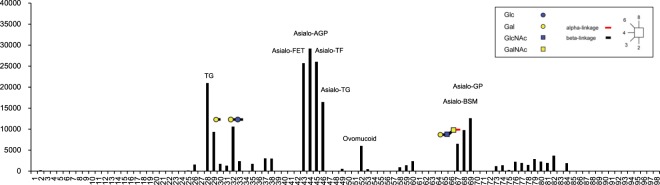
Glycoconjugate microarray analysis of the sugar-binding specificity of AJLec. Cy3-labeled AJLec was used to probe the microarray. The net intensity value of each spot represents the signal intensity minus the background value (Cy3-labeled BSA). The oligosaccharides are indicated by numbers that correspond to those shown in Supplementary Table S3.

To investigate the binding specificity of AJLec for complex carbohydrates, a glycoconjugate microarray analysis was performed (Fig. 1 and Supplementary Fig. S3). Using a series of complex oligosaccharides, this analysis revealed significant binding to the three types of carbohydrates containing terminal galactose with β1-4 linkage (entry nos 29, 32, 67). In contrast, carbohydrates containing interior (non-terminal) galactoses showed negligible binding ability (entry nos 2, 5–8, 12, 14, 19–24, 31, 42, 72). This implies that AJLec specifically binds to β-linked terminal galactose residues in complex carbohydrates. With this binding activity, a common tendency in glycoprotein activity was confirmed. AJLec showed significant binding activity towards all of the asialoglycoproteins (entry nos 43–46, 68, and 69), which are notated as “Asialo-” in Fig. 1. In contrast, no clear binding activity was observed for agalactoglycoproteins (entry nos 49–51) with no terminal galactose residues (nos 25–27). Non-treated glycoproteins with significant binding activity (entry nos 28, 52) are modified with complex-type N-glycans. Structures of the complex-type N-glycans modified on mature proteins are heterogeneous in most cases, and some types of complex-type N-glycans contain β-linked galactose residues in terminal positions^11,12^. Therefore, the binding activities for these glycoproteins (entry nos 28, 52) could also be accomplished by binding to β-linked terminal galactose residues in their carbohydrates.

In terms of binding to GalNAc-containing carbohydrates, no definite binding to any carbohydrates, including GalNAc and excluding terminal galactose residues, was shown (entry nos 7, 8, 31, 39–42, 58, 61–64, 70–72). This indicates that AJLec does not bind to GalNAc residues in complex carbohydrates, which agrees with the results of the hemagglutination inhibition assay.

### Amino acid sequencing of AJLec

Edman degradation of AJLec did not yield any N-terminal amino acids, suggesting that this lectin had a blocked amino terminus. Therefore, to obtain information on the internal sequence, the protein was cleaved with CNBr at methionine residues. Several peptides were obtained after separation by reverse-phase high-performance liquid chromatography (HPLC) (Supplementary Fig. S4). A sequence analysis of the three peptides yielded 27, 17, and 34 amino acid residues: peak fraction numbers 2 and 3, FPKTGATYYLDPYVIKNRFYGVQGYNA; peak fraction numbers 5 and 4–2, ESDNSGKVCADVFGYFV; and peak fraction number 4–1, SPELVLQHGCNSPSDYIGPDSQLRVWYGEDLYNT.

### cDNA cloning and sequence analysis of AJLec

**Figure 2:**
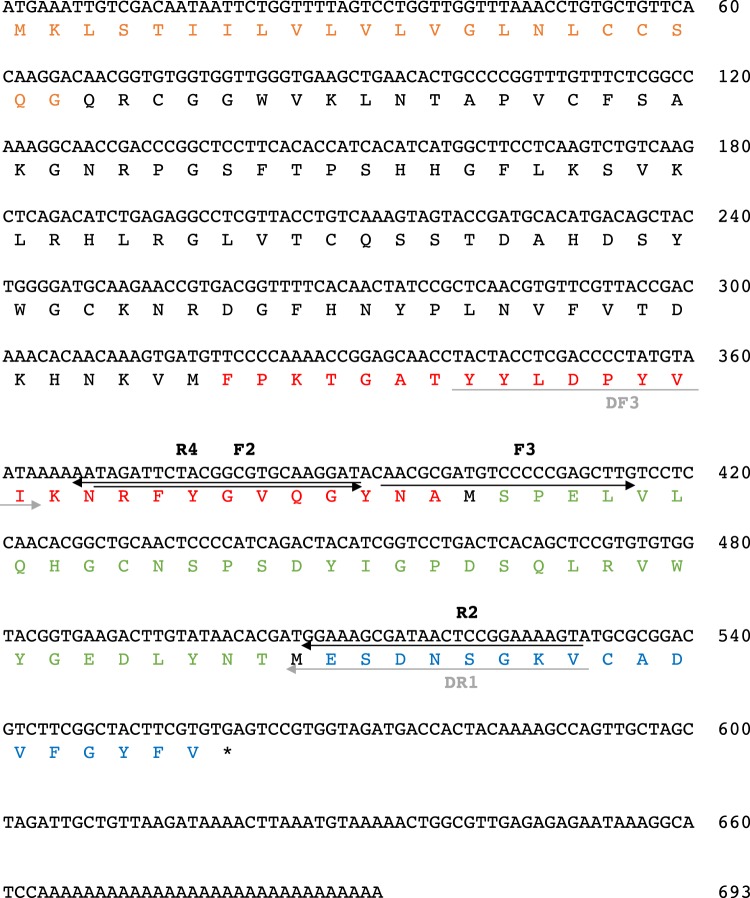
The nucleotide and deduced amino acid sequence of AJLec. A partial amino acid sequence determined from the purified protein presented in colored letters, with fraction numbers 2 and 3 colored red, fraction number 4–1 colored green, and fraction numbers 4–2 and 5 colored blue. The primers used for PCR are indicated by horizontal arrows.

mRNA purified from *A. japonica* was used for cDNA synthesis. Amplification of the DNA fragment was performed by PCR using the degenerate primers DF3 and DR1, designed from two partial sequences of AJLec. As a result, a fragment of approximately 200 bp was amplified, and its nucleotide sequence was determined. Primers F2 and F3 (nested primers) were then designed on the basis of this sequence and used for 3′-RACE to determine the 3′-terminal sequence of the cDNA. Additional amplification of the cDNA was performed by 5′-RACE using the primers R2 and R4, resulting in a complete AJLec cDNA sequence, as shown in Fig. 2. The open reading frame of AJLec consists of 558 bp, corresponding to 186 amino acid residues. The 22 N-terminal amino acid residues were assumed to be the signal sequence, and the mature protein was found to contain 164 amino acid residues with a molecular mass of 18,491 Da.

### Comparison of the AJLec amino acid sequence and that of other proteins

A Blast search for homologous proteins of AJLec revealed seven predicted proteins from *Nematostella vectensis* (starlet sea anemone), whose whole genome has only been sequenced in sea anemone species, with 28–49% homology (Supplementary Fig. S5). In addition, 29 kinds of predicted proteins from *Stylophora pistillata* (Smooth cauliflower coral), whose whole genome sequence was recently decoded^13^, have homology with that of AJLec with 29–40%. Their expression has not been confirmed in these organisms. This suggests that proteins homologous to AJLec exist in sea anemone and coral species only. To elucidate its folding and the structural basis for its specificity, a structural analysis of the AJLec-lactose complex was carried out as described below.

### Mass spectrometric (MS) analysis of N-terminal modifications

A peptide mass fingerprinting (PMF) analysis of the MS spectrum from a Pam-AJLec digest identified five major ions (m/z 1221.64, m/z 1503.80, m/z 1738.91, m/z 2036.04, and m/z 2,875.40, shown in gray italics) as internal peptides and a minor ion (m/z 1190.56, shown in gray) in the C-terminal peptide. The most prominent ion (m/z 987.50, shown in bold), which was not assigned by PMF, was 17 Da less than that of the sequence mass (m/z 1,004.51) of the theoretical N-terminal peptide (QR[Pam-C]GGWVK), suggesting that the N-terminal residue of mature AJLec is pyroglutamylated (Supplementary Fig. S6A). This assumption was confirmed by MS/MS analysis (Fig. S6B). The MS/MS spectrum of the precursor ion (observed m/z 987.50 in Supplementary Fig. S6A) matched the N-terminal structure of Pyro-QR[Pam-C]GGWVK (theoretical m/z 987.48), and the spectrum was assigned to the b-ion series (b3-b7) with a significant probability-based MASCOT score (*p* < 0.05; peptide score, 27; expected value, 0.0023).

### Crystal structure of AJLec

**Figure 3:**
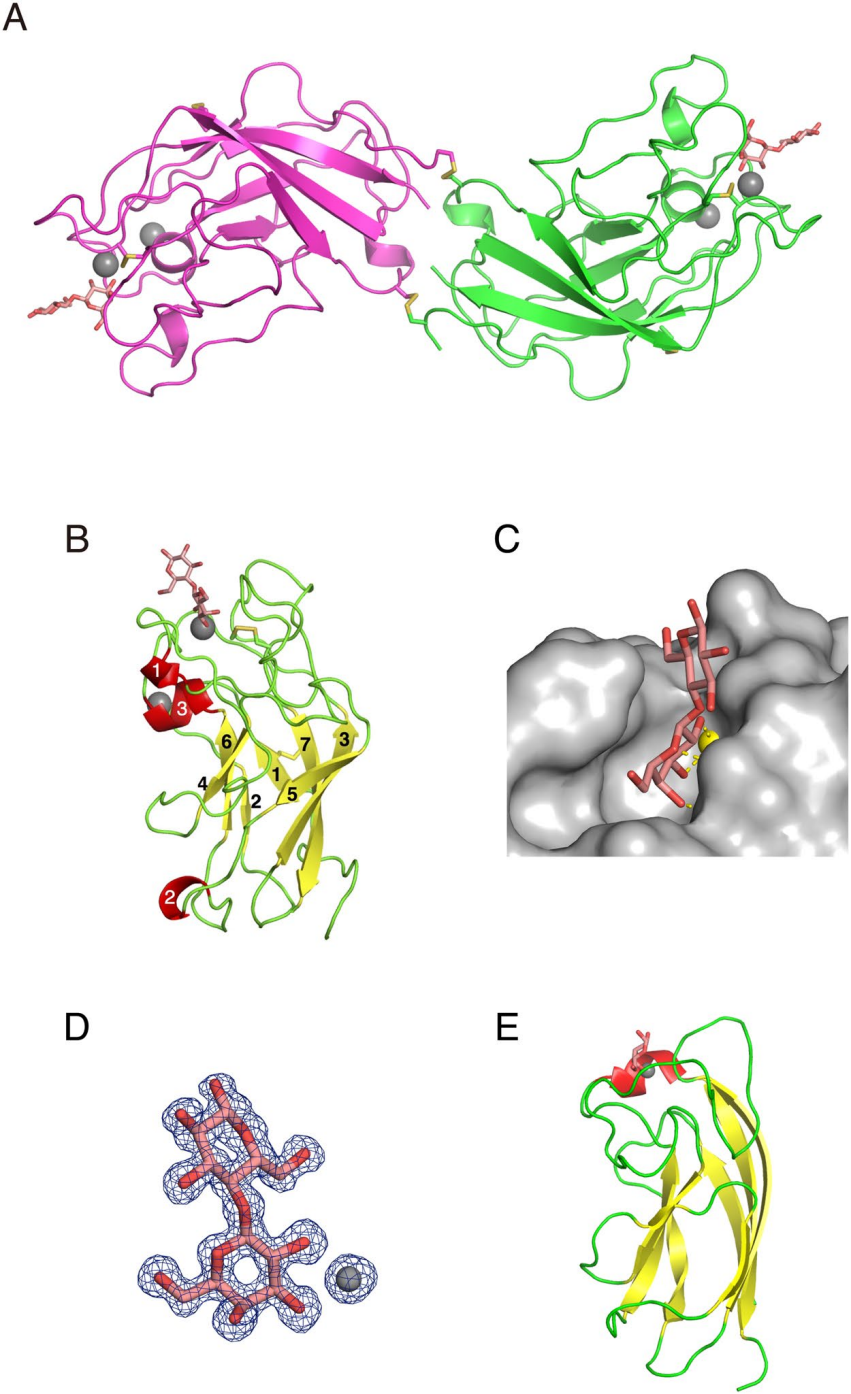
Crystal structure of AJLec. The crystal structure is shown in ribbon mode. (**A**) Dimer structure of AJLec. Each protomer is shown in a different color. Lactose molecules that are bound to AJLec are shown as orange stick figures. Ca^2+^ ions are shown as gray spheres. S-S bonds are shown in yellow. Each protomer of the dimer structure shows twofold crystallographic symmetry. (**B**) Protomer structure of AJLec. Yellow and red, β-sheets and α-helices, respectively; they are labeled in the order of appearance. (**C**) Surface figure at lactose-binding site. Lactose molecule and Ca^2+^ ion are shown as stick model and yellow sphere, respectively. The yellow dotted lines denote hydrogen and coordinate bonds with carbohydrates. (**D**) *F*_o_ − *F*_c_ omit electron density map (blue) for lactose and Ca^2+^ ion bound to AJLec. The contour level of the omit maps is 3σ. (**E**) Protomer structures of the PA-IL/galactose complex (PDB code: 4AL9). A galactose molecule that is bound to PA-IL is shown as an orange stick figure. The other color representations are the same as described in panel B.

The crystal structure of AJLec was solved by sulfur single-wavelength anomalous diffraction phasing with an in-house Cu Kα source (S-SAD with Cu Kα). The S-SAD with Cu Kα method utilizes the slight anomalous dispersion effect of sulfur that can cause difficulty in the phase determination of protein structures. To overcome this difficulty, we enforced high-redundancy data collection in a diffraction experiment and non-crystallographic symmetry (NCS) averaging in phase improvements. Electron density maps after the phase improvements yielded high enough quality images for tracing all residues of AJLec. The crystal structure of AJLec in complex with lactose was determined in the orthorhombic space group *P*2_1_2_1_2_1_ with unit cell parameters *a* = 38.1 Å, *b* = 79.7 Å, and *c* = 106.0 Å. The final model was refined to *R* and *R*_free_ values of 13.6% and 16.9% for all data in the resolution range of 63.7–1.20 Å. Two polypeptide chains were found to be present in the asymmetric unit, and a total of 164 amino acid residues per polypeptide chain were observed. There are two disulfide bonds at Cys3 and Cys122 between the two chains in the asymmetric unit (Fig. 3A). Each of the protomers were tightly associated with the buried area of 461 Å^2^, including hydrogen bonds and hydrophobic interactions, in addition to the disulfide bonds. Therefore, the dimer structure, formed due to the formation of the disulfide bonds, was supposed to be a functional dimer in solution. The dimer structure forms a helical-bar like shape, where lactose binding sites are located on both of its termini.

The protomer structure of AJLec was composed of a core structure with seven β-sheets and three surrounding helices and loops (Fig. 3B, Supplementary Fig. S7). The seven β-sheets formed small jelly-roll type β-sandwich folds. A sugar-binding site was a little distant from the core structure of the β-sheets and formed by residues on helix 3 and loops between sheets 3 and 4, sheet 4 and helix 1, and helix 3 and sheet 7. These residues configurated a groove, where the lactose molecule fits (Fig. 3C). A lactose molecule in the site was bound to a Ca^2+^ ion, where its glucose residue was quite solvent exposed whilst its galactose residue was mainly buried in the binding site groove. In addition, one more Ca^2+^ ion was bound at a position, which was 10 Å apart from the sugar-binding site. The second binding site for the Ca^2+^ ion was composed of residues, Asn21, Asp142 Asn145, and Glu148, that was supposed to stabilize the overall structure (Fig. 3A,B, Supplementary Fig. S8). Clear electron density maps were shown for nearly all of residues, lactose molecules, and Ca^2+^ ions (Fig. 3D). In agreement with the results of MS analysis of the N-terminal peptide, an atomic model of pyroglutamic acid was fitted to the electron density map on the N-terminal residue (Supplementary Fig. S9). Internal disulfide bonds between Cys15 and Cys156 and between Cys48 and Cys61 were located in the β-sandwich fold and loop regions, respectively.

**Figure 4:**
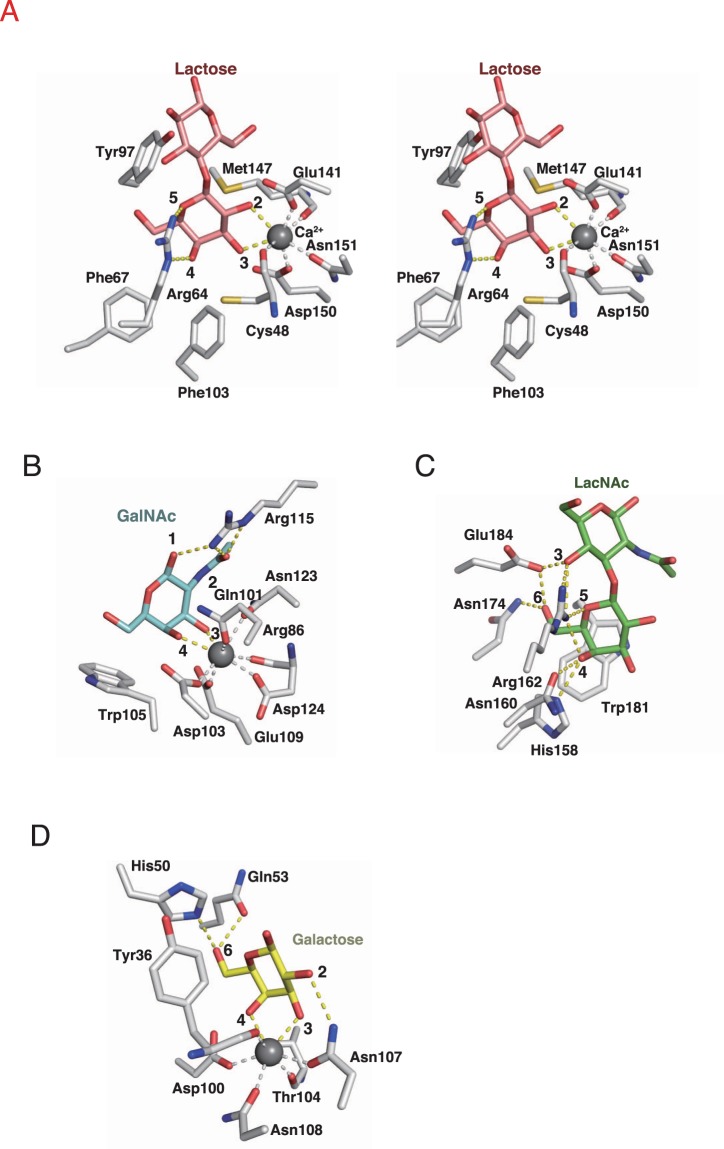
Residues involved in the binding of specific carbohydrates to AJLec, CEL-I, galectin-3, and LecA. Stick models for the interactions of lactose with AJLec (**A**) (stereo view), GalNAc with CEL-I (**B**) (PDB code: 1WMZ), LacNAc with human galectin-3 (**C**) (PDB code: 4XBN), and LecA with galactose (**D**) (PDB code: 1OKO). Ca^2+^ ions are indicated in gray spheres. Lactose, GalNAc, LacNAc, Galactose, and the residues are shown in stick figures. The yellow dotted lines denote hydrogen and covalent bonds with carbohydrates. The gray dotted lines denote coordinate bonds between Ca^2+^ ions and residues. Residues without dotted lines have hydrophobic interactions with the sugars.

β-galactose residue in lactose is held by two coordinate bonds with Ca^2+^ ion and two hydrogen bonds with side chain nitrogen atoms of Arg64; hydroxyl groups at positions 2, 3, and 4, and oxygen at position 5 in the β-galactose residue form hydrogen and coordinating bonds in the site (Fig. 4A). The binding of β-galactose residues was further stabilized by hydrophobic interactions between the hydrophobic face of the β-galactose residue in the lactose and side chains of Phe67, Tyr97, and Phe103. The sugar residue is also engaged in a hydrophobic contact with the side chain of Met147. In addition, the Ca^2+^ ion coordinately bound sidechain oxygen atoms of Glu141, Asp150, and Asn151, and main chain oxygen atoms of Met147 and Cys48, present in these sites. Cys61 also participate in the shape of the binding site (Supplementary Fig. S10).

Glucose residue in lactose is located near Tyr97, and showed hydrophobic interactions with Met147 (Supplementary Fig. S10).

## Discussion

AJLec was purified from *A. japonica* and identified as a Ca^2+^-dependent and galactose-specific lectin (18.5 kDa) forming a disulfide-bonded dimer. Hemagglutination inhibition assay, ITC analysis, and glycoconjugate microarray revealed its strict specificity for β-linked terminal galactose, but not for GalNAc. This specificity of AJLec cause it to keep its distance from many known galactose-binding lectins showing the ability to bind to GalNAc or a low ability to bind to galactose. C-type lectins with QPD (Gln-Pro-Asp) motifs and galectin families are major examples of galactose-binding lectin families^14^. For example, CEL-I, a C-type lectin from *Cucumaria echinata*, shows affinity for GalNAc and galactose^15^. Galectins show a high binding constant for LacNAc (Galβ1–4GlcNAc)^16^ (sheep galectin-1; 46.4 × 10^3^ K_a_)^17^, whereas that for galactose is relatively low (sheep galectin-1; 0.05 × 10^3^ K_a_)^17^. Peanut agglutinin PNA shows significant affinity for Galβ1-3GalNAc (49.0 × 10^3^ K_a_), which is much stronger than that for galactose (0.69 × 10^3^ K_a_)^18^. These reported galactose-binding lectins have a comparatively large specificity for more than one monosaccharide structure or strict specificity for particular structures of complex carbohydrates. To this date, AJLec was only found present in sea anemone and coral species throughout the course of evolution; therefore, the characteristics of AJLec should be useful for the specific environment of the sea anemone and coral, and its immune system.

The primary structure of AJLec was not homologous with that of any known lectins. Accordingly, AJLec was assumed to be a novel type of lectin with a unique structure and specificity. In the sequence alignment of AJLec with homologous proteins from *N. vectensis* and *S. pistillata*, all the residues coordinating Ca^2+^ ion in the sugar-binding site are conserved. This indicates that the binding of Ca^2+^ ion is also crucial for function of the homologous proteins. On the other hand, residues interacting with galactose and coordinating with the second Ca^2+^ ion are not conserved partly in the homologous proteins. This indicates that the binding specificity in the homologous proteins would differ from that in AJLec, and coordination for the second Ca^2+^ ion would not be formed in the homologous proteins (Supplementary Fig. S5).

The crystal structure of AJLec was solved by S-SAD phasing with an in-house Cu Kα source (S-SAD with Cu Kα). This method is extremely beneficial in preparing crystals and in terms of facilities. Namely, it does not require the preparation of any heavy atom derivatives or any facilities other than a typical in-house X-ray diffraction device. However, because of technical difficulties, with weak anomalous signals from S atoms at a wavelength of 1.54 Å, reported structures solved by the method have been rare until now. To overcome the problem of the weak anomalous signals, the S-SAD phasing was used to focus on low energy X-ray diffraction measurements in synchrotron beamlines^19^. For instance, of the few protein structures solved by the S-SAD with Cu Kα method, CGL1^7^ (our previous report) and AJLec (this report) are first and second lectin structures, respectively, to be solved. In the determinations of both of these structures, we overcame the difficulties of the technique for high-redundancy data collection and NCS averaging for phase improvement. These practical accomplishments also showed that this technique does not require higher resolution data than that collected from crystals of moderate lectins (~1.5 Å resolution). This indicates that the S-SAD with Cu Kα method could be adapted for routine structural determinations of lectins and many other oligomeric proteins.

The crystal structure of AJLec showed a disulfide-bonded dimer with a unique helical-rod like shape. The protomer structure indicated seven β-sheets forming a small jelly-roll type β-sandwich fold. A structure-based search using the DALI server^20^ showed some structural similarity with PA-IL (LecA) in *Pseudomonas aeruginosa* (root mean square deviation; 3.6 Å). PA-IL has similar characteristics as AJLec in its folds (small jelly-roll type β-sandwich folds), function (galactose-binding in a Ca^2+^ ion-dependent manner), and positions of sugar-binding sites in protomer structures (Fig. 3E)^21^. On the other hand, there was no homology observed in amino acid sequences, structures in sugar-binding sites, and quaternary structures (AJLec, dimer; PA-IL, tetramer) between AJLec and PA-IL. In addition, details of the binding specificities of the lectins differ, at least in part (AJLec, terminal galactose, but not GalNAc; PA-IL; galactose and GalNAc)^22^. Hence, the folding similarity did not evolve from a common ancestor, but was the result of convergent evolution of the lectins.

Sugar-binding sites were found to be located on both termini of the dimeric structure. The orientation of the sites showed a pattern common in dimeric lectins that indicated agglutination activity against polysaccharide-coated pathogenic bacteria. Pyroglutamic modification in N-terminal residues of AJLec was identified by mass analysis and confirmed by crystallography. This modification has been identified in a variety of proteins with known structural and functional roles^23^. The N-terminal residues that interact for dimerization are distant from the sugar-binding site. Therefore, the modification in AJLec should not affect its sugar-binding activity, but could be related to the retention of the structure, stability, or secretion of the protein, although details of its function in AJLec are unclear.

In the sugar-binding site of AJLec (Fig. 4A), the interactions, especially those for coordination and hydrogen bonding, have not been observed in other known lectins. The interactions enable strict specificity for β-linked terminal galactose residues, as revealed in the glycoconjugate microarray analysis. In the case of binding with α-linked terminal galactose residues at the position, steric hindrances between adjacent carbohydrate and residues of Met147 and Tyr97 may arise, that would block the binding (Supplementary Fig. S10). As another interesting characteristic of AJLec specificity, GalNAc binding activity was not observed in the hemagglutination inhibition assay or glycoconjugate microarray analysis. The 2′-OH position of the galactose residue formed a coordinate bond with the Ca^2+^ ion in the lactose-AJLec complex. Therefore, the Ca^2+^ ion could not form a coordinate bond with GalNAc, in which the 2′-OH residue was replaced by an N-acetyl residue. This would be the structural basis for the inability of AJLec to bind to GalNAc. This shows the importance of interactions between the 2′-OH group in galactose and residues in AJLec, whereas many other galactose-binding lectins do not show this interaction (Fig. 4B,C)^24,25^. For examples, of lectins in known major lectin families, the importance of interactions only with 3′ and 4′-OH in galactose residues is apparent in C-type lectins with QPD motifs^24^. Similarly, the importance of interactions only with 4′ and 6′-OH in galactose residues has been noted for galectins^14^. Therefore, many galactose-binding lectins also show binding specificity for GalNAc.

In the comparison of AJLec and known calcium-binding lectins, C-type lectin and LecA (PA-IL), the known lectins were found to form coordinate bonds with Ca^2+^ ion to bind to target carbohydrates, but their coordination patterns in carbohydrates definitely differ from that of AJLec (Fig. 4A,B,D). Most C-type lectins with galactose specificity have QPD motifs for coordination bonding to the Ca^2+^ ion, and the Ca^2+^ ion also has coordinate bonds to hydroxyl groups at positions 3 and 4 of the galactose/GalNAc residues. LecA also has coordinate bonds in the same manner as that of the C-type lectins, while it has not any corresponding motif. In contrast to the lectins, AJLec has coordinate bonds to the hydroxyl groups at positions 2 and 3 of the galactose residue. If galactose residues in the AJLec complex were replaced with GalNAc, steric hindrance would occur, resulting in a failure to bind. It has been reported recently that Lectin A (PIIA), which has homology with LecA, binds α-linked terminal galactose but not GalNAc^26^. Coordination pattern and interactions in Lectin A for α-galactoside are similar to that in LecA, while additional hydrogen bond with hydroxyl group at positions 2 in α-galactoside was confirmed in Lectin A. It would be a structural basis for an abolishment of binding in the case of GalNAc.

In summary, novel galactose-binding lectin AJLec was purified from *A. japonica*. AJLec shows a unique specificity for galactose monomers and β-linked terminal-galactose residues, which differs from that of known galactose-binding lectins. The primary structure of AJLec was not homologous with that of any known lectins, indicating that this was a new type of lectin. The structural analysis of AJLec revealed a disulfide-bonded dimer structure with a unique helical-rod-like shape. The protomer structure of AJLec showed a jelly-roll type β-sandwich fold and a sugar-binding site containing a Ca^2+^ ion and bound-lactose molecule in the AJLec/lactose complex. The structure of the binding site and coordination pattern for the Ca^2+^ ion, which are dissimilar to those of any other type of lectin, conferred the specificity for β-linked terminal-galactose residues in carbohydrates. This characteristic of AJLec could lead to its use as a research tool and in medical procedures, because AJLec would have broad utility in profiling structures of complex carbohydrates^27,28^. In addition, the S-SAD with Cu Kα method used to solve the structure of AJLec might be routinely applied for structural determinations of lectins and many other oligomeric proteins.

## Experimental Procedures

### Animals

Sea anemones (*A. japonica*) from the Ariake Sea were purchased from a local dealer. Their bodies were stored at −20 °C.

### Purification of AJLec from *A. japonica*

AJLec was purified from *A. japonica* by affinity chromatography using a lactose-immobilized Cellufine (Seikagaku Kogyo, Tokyo, Japan) column prepared as previously reported^29^. Frozen bodies of *A. japonica* (300 g) were suspended in 600 mL of TBS-Ca^2+^ (Tris-buffered saline; 10 mM Tris-HCl pH 7.6, 150 mM NaCl, and 10 mM CaCl_2_), and disrupted in a blender, followed by centrifugation at 9,500 × *g* for 30 min. The supernatant was applied to the lactose-Cellufine column (10 mL) pre-equilibrated with TBS-Ca^2+^. After washing with this buffer, AJLec was eluted with 100 mM lactose in TBS. The protein was further purified by gel filtration on a HiLoad 26/60 Superdex 200 prep grade column (GE Healthcare) equilibrated with TBS and then eluted at a flow rate of 2.5 mL/min using an ÄKTAprime Plus apparatus (GE Healthcare). The eluate was dialyzed with TBS, concentrated to 1.0–10 mg/mL, and then used for subsequent experiments, except for crystallization. For crystallization of the lactose complex with AJLec, the purified protein solution, including 100 mM lactose and 1 mM CaCl_2_, was concentrated to 9 mg/mL.

### Lectin activity assays

Standard hemagglutination assays were performed using rabbit, horse, sheep, and bovine erythrocytes. The assays were performed in 96-well U-bottom plates with serial twofold dilutions of a 50-μL lectin solution with an equal volume of TBS-Ca^2+^ buffer. Then, 50 μL of 5% (v/v) erythrocyte suspension in the same buffer was added to each well. The plates were incubated for 1 h, and the formation of a sheet (an agglutination-positive result) or a dot (an agglutination-negative result) was monitored. The results are expressed as the minimal protein concentration required to produce visible agglutination. To examine the effect of divalent ions on AJLec hemagglutination activity, AJLec dialyzed with TBS buffer (0.01 M Tris-HCl and 0.15 M NaCl, pH 7.5) were used for the hemagglutination assay. The lowest divalent ion concentration resulting in agglutination was determined by twofold serial dilution of solutions at a 25-mM initial concentration.

### ITC

Sugar specificity and thermodynamic parameters were measured by ITC using a MicroCal iTC_200_ microcalorimeter (GE Healthcare). The titration was carried out at 25 °C, with the ligand and AJLec dissolved in TBS-Ca^2+^. The measurements were performed in a cell with a volume of 200 μL with 20 injections of 2-μL ligands at 2-min intervals. Protein and ligand solutions were used at concentrations of 0.56 mM (10 mg/mL) and 5.6 mM, respectively. Control experiments were carried out to measure the ligand dilution-related temperature, which was subsequently subtracted from the ligand binding thermograms.

### Glycoconjugate microarray

The sugar-binding specificity of AJLec for complex oligosaccharides was analyzed using a glycoconjugate microarray as described in a previous report^30^. Briefly, glycoproteins and glycoside-polyacrylamide conjugates were dissolved in a spotting solution (Matsunami Glass, Osaka, Japan) and spotted on a microarray-grade epoxy-coated glass slide (Schott AG) using a non-contact microarray-printing robot (MicroSys 4000; Genomic solutions). AJLec was labeled with Cy3-NHS ester (GE healthcare). The Cy3-labeled AJLec solution (10 μg/ml in TBS-Ca^2+^) was incubated with the glycoconjugate microarray at 20 °C overnight. After washing, images were immediately acquired using a Bio-REX Scan 200 evanescent field-activated fluorescence scanner (Rexxam, Osaka, Japan).

### Chemical cleavage, separation, and sequencing of peptides

The purified AJLec was reduced with tri-*n*-butylphosphine in 7 M guanidine-HCl, 10 mM EDTA, and 0.5 M Tris-HCl (pH 8.5) and pyridylethylated by treatment with 4-vinyl pyridine at 25 °C for 4 h in the dark. The resulting pyridylethylated AJLec was chemically cleaved at methionyl bonds with 1% CNBr in 70% (v/v) formic acid at 25 °C for 24 h by the method described by Gross^31^. The peptides generated by CNBr cleavage were separated by reverse-phase HPLC using HITACHI model L-6200 and L-4200 liquid chromatographs on a Wakosil-II 5C18 AR column (4.6 × 100 mm, Wako Pure Chemical Industries Ltd., Osaka, Japan). The column was equilibrated with solvent A (0.1% trifluoroacetic acid; TFA), and the peptides were eluted at a flow rate of 1 mL/min using a linear gradient of 0–100% solvent B (acetonitrile/water/TFA, 80:20:0.1 [v/v/v]) at room temperature. Amino acid sequences of the separated peptides were determined using a PPSQ-21 protein sequencer (Shimadzu).

### cDNA cloning of AJLec

The bodies of *A. japonica* were flash frozen in liquid nitrogen and ground to powder form. Total RNA was extracted with Isogen solution (Nippon Gene, Tokyo, Japan). Poly(A) RNA was collected using an Oligotex-dT30 mRNA purification kit (Takara, Otsu, Japan), and cDNA was synthesized using a SMARTer cDNA Cloning Kit (Clontech). The desired DNA fragment was amplified by polymerase chain reaction (PCR) using two degenerate primers: DF3, 5′-TAYTAYYTIGAYCCITAYGTIATIAA-3′, and DR1, 5′-AARTAICCRAAIACRTCIGCRCA-3′. This DNA fragment was cloned into a pTAC-2 vector using *E. coli* JM109 cells (Clontech) and sequenced with an ABI PRISM 3130 Genetic Analyzer (Applied Biosystems). The amino acid sequence deduced from this DNA fragment agreed with the peptide sequences determined from the purified protein. Therefore, 3′- and 5′-rapid amplification of cDNA ends (3′-RACE and 5′-RACE) was preformed using primers F2, F3, R2, and R4, with F3 and R4 designed from this region using a SMARTer cDNA Cloning Kit and used for a nested PCR.

### Bioinformatics analysis

Online sequence homology searches (www.ncbi.nlm.nih.gov) were performed using the BLASTP algorithm in non-redundant databases of the National Center for Biotechnology Information (NCBI)^32^. Alignment of the sequences was performed using Clustal Omega software^33^.

### Crystallization

Crystallization of AJLec was performed using the vapor diffusion method. Two to four microliters of the protein solution in TBS containing 100 mM lactose and 1 mM CaCl_2_ was mixed with the same volume of a reservoir solution (200 mM NaCl, 100 mM HEPES [pH 7.5], and 30% (w/v) polyethylene glycol 3350) and subjected to vapor diffusion at 20 °C.

### Data collection, structure determination by S-SAD, and refinement

The AJLec data set (S-SAD) was collected in-house (MicroMax007 & R-AXIS IV_++_ [RIGAKU]), whereas those of AJLec (high-resolution) were collected using a beamline BL-1A at the Photon Factory (KEK, Tsukuba, Japan). Crystals of AJLec (S-SAD) and AJLec (high-resolution) were frozen at 120 K and 95 K, respectively, before data collection. The AJLec data set (S-SAD) was processed and scaled using MOSFLM^34^ and SCALA software^35^, respectively, and that from AJLec (high-resolution) was processed and scaled using HKL2000^36^. The AJLec crystal data sets belonged to space group *P*2_1_2_1_2_1_, with two molecules per asymmetric unit.

After failing in initial screens to generate heavy atom derivatives by soaking heavy atoms in AJLec crystals, we tried to determine the AJLec structure using the S-SAD with Cu Kα method. To overcome the difficulty of calculations with a slight anomalous dispersion effect from sulfur, we enforced high-redundancy data collection to obtain statistically processed data with sufficient accuracy for S-SAD measurements. The collected data set, AJLec (S-SAD), was used for phase calculation in PHENIX^37^. Phase improvement by density modification, including NCS averaging, was also performed in PHENIX^37^. Electron density maps, obtained after phase improvements, including NCS averaging, were of sufficiently high quality to trace all residues in AJLec. The structure was built using COOT software^38^ and refined in Refmac^39^, with 5% of the data set aside as a free set. During subsequent refinement, the AJLec (S-SAD) data set was replaced by the AJLec (high-resolution) data set and anisotropic refinement was enforced. The lactose models were fit into the carbohydrate-binding sites according to the difference electron density map. The refinement statistics are shown in Table 3. All figures were produced using PyMOL software (http://www.pymol.org). Secondary structure assignment was performed in DSSP^40^, and the interface surface area and assemblies of AJLec were calculated using PISA^41^.

**Table 3:** Data collection and refinement statistics.

Crystal type	S-SAD	High-resolution
Data collection and processing statistics		
Beam line	MicroMax007HF & R-AxisIV++	PF BL-1A & Eiger X4M
Space group	*P*2_1_2_1_2_1_	*P*2_1_2_1_2_1_
Unit cell dimension (Å)		
*a* (Å)	38.0	38.1
*b* (Å)	79.7	79.7
*c* (Å)	106.2	106.0
Wavelength (Å)	1.5418	1.1000
Resolution (Å)	28.8–1.50 (1.58–1.50)	63.7–1.20 (1.23–1.20)
Total reflections	1,398,189	1,120,545
Unique reflections	48,526	101,402
*I*/*σI*	32.7 (4.2)	25.6 (6.7)
Redundancy	28.8 (17.5)	11.1 (8.0)
Completeness (%)	92.4 (56.1)	99.7 (96.0)
*R*_merge_^*a*^ (%)	6.5 (46.5)	5.6 (24.3)
Number of S-atoms	18	
Refinement statistics		
Resolution		63.7–1.20
Protein atoms		2616
Ligand atoms		65
Water molecule		400
*R*_work_/*R*_free_(%)		14.2/17.6
Root mean square deviations		
Bond lengths (Å)		0.016
Bond angles (°)		1.887
Ramachandran statistics (%)		
Residues in favored region		93.9
Residues in allowed region		6.1
Residues in outlier region		0

Numbers in parentheses are for the highest shell. ^*a*^*R*_merge_ = 100Σ|*I* − <*I*>|/Σ *I*, where *I* is the observed intensity and <*I*> is the average intensity from multiple observations of symmetry-related reflections.

### Mass spectral analysis of N-terminal modifications

Cysteine residues of AJLec were subjected to Cys-S-β-propionamidation using the method of Sechi and Chait with slight modifications^42^. In brief, 20 µL of AJLec (approximately 1 mg/mL) was added to 20 µL of 2× SDS-sample loading buffer for Cys alkylation (125 mM Tris-HCl (pH 8.5), 20% glycerol, 4% SDS, 10% 2-mercaptoethanol, and 0.0025% bromophenol blue), and the protein solution was incubated at 95 °C for 10 min. Twenty microliters of 7 M acrylamide was added to the reduced protein solution and held for 1 h at room temperature. The resulting propionamidated (Pam)-AJLec solution (20 µL/well) was applied to SDS-PAGE^43^, and the protein band was stained with Coomassie Brilliant Blue. An in-gel enzymatic digestion was performed according to a method described by Shevchenko *et al*.^44^. The gel section was excised from the Pam-AJLec band using a gel picker (1.8-mm diameter, Anatech, Japan), destained twice with 200 µL of 30% acetonitrile in 25 mM NH_4_HCO_3_, dehydrated with 100 µL of acetonitrile, and digested in-gel with lysyl endopeptidase (mass spectrometry grade, Wako Pure Chemical Industries, Japan) solution (20 µg/mL in 50 mM Tris-HCl, pH 8.8) at 37 °C for 16 h. Peptide extraction from the gel and sample loading onto a matrix-assisted laser desorption/ionization (MALDI) target plate was performed as described by Yamaguchi^45^. MS and MS/MS spectra were obtained using a matrix-assisted laser desorption/ionization (MALDI) quadruple-ion trap (QIT) time-of-flight (TOF) mass spectrometer (AXIMA Resonance, Shimadzu, Japan) with 2, 5-dihydroxybenzoic acid (DHBA, Shimadzu, Japan) as the matrix in positive mode. MALDI-QIT-TOF mass spectra were externally calibrated using human angiotensin II (m/z 1,046.54) and human ACTH fragment 18–39 (m/z 2,465.20) in a ProteoMass Peptide and Protein MALDI-MS Calibration Kit (Sigma-Aldrich, MO, USA). Modification of the N-terminal peptide was analyzed by PMF and an MS/MS ion search using MASCOT ver. 2.3 (Matrix Science, London, UK) with an original database (JGL1 and other 7258 protein sequences; 2,279,877 residues) in our own MASCOT server. Search parameters used for PMF were the following: enzyme, Lys-C; fixed modifications, propionamide (C); variable modifications, oxidation (HW and M); mass values, monoisotopic; peptide mass tolerance, +/−0.3 Da; peptide charge state, 1+; and maximum number of missed cleavage sites, 2. Search parameters used for the MS/MS ions search were as follows: enzyme, Lys-C; fixed modifications, propionamide (C); variable modifications, oxidation (HW and M) and Gln → pyro-Glu (N-term Q); mass values, monoisotopic; peptide mass tolerance, +/−0.3 Da; fragment tolerance, +/−0.2 Da; max missed cleavages, 2. A protein score (PMF) > 51 and individual ion score (MS/MS ions search) >14 were considered significant (*p* < 0.05).

### Accession numbers

The atomic coordinates and structural parameters of AJLec were deposited in the Protein Data Bank, www.pdb.org (PDB code: 6A56). The nucleotide sequence of AJLec was deposited in DDBJ/EMBL/GenBank (accession number: LC339819). This work was performed under the approval of the Photon Factory Program Advisory Committee (proposal no. 2014G515).

## Electronic supplementary material

Supporting Information
