# Supplementary material for: Identification, Characterization, and X-ray Crystallographic Analysis of a Novel Type of Lectin AJLec from the Sea Anemone Anthopleura japonica
Source: Sci Rep. 2018 Aug 1;8:11516. doi: 10.1038/s41598-018-29498-0 (PMC6070535; doi:10.1038/s41598-018-29498-0)
Supplement: Supplementary file 1 — Supporting Information [file 41598_2018_29498_MOESM1_ESM.pdf]

Supporting Information

Identification, Characterization, and X-ray Crystallographic Analysis of a Novel Type of Lectin AJLec from the Sea Anemone *Anthopleura japonica*

Hideaki Unno, Azusa Nakamura, Shingo Mori, Shuichiro Goda, Kenichi Yamaguchi, Keiko Hiemori, Hiroaki Tateno & Tomomitsu Hatakeyama

Supporting Information includes the following:

Table S1. Hemagglutinating activities of AJLec.
Figure S1. Purification of AJLec.
Figure S2. Titration calorimetry results for AJLec with galactose and lactose.
Figure S3. List of oligosaccharides in the glycoconjugate microarray analysis.
Figure S4. Elution profiles from high-performance liquid chromatography of the peptides.
Figure S5. Alignment of the deduced amino acid sequences of AJLec and proteins of *Nematostella vectensis*.
Figure S6. Mass spectrometric analysis of the N-terminal peptide from the lysyl endopeptidase digest of Pam-AJLec.
Figure S7. Topological diagram illustrating the secondary structural elements of AJLec.
Figure S8. Second binding-site for Ca^{2+} ion in AJLec.
Figure S9. N-terminal modification of AJLec.
Figure S10. Stereo Figure of lactose and adjacent residues in AJLec

Table S1. Hemagglutinating activities of AJLec.

Erythrocytes	Hemagglutinating activity (μg/mL)
Rabbit	0.65
Horse	36
Sheep	21
Bovine	18
**Divalent cation**	**Hemagglutinating activity (mM)**
No divalent cation	N. A.
Ca^{2+}	0.1
Mg^{2+}	>25
Mn^{2+}	>25

N.A.: No agglutination

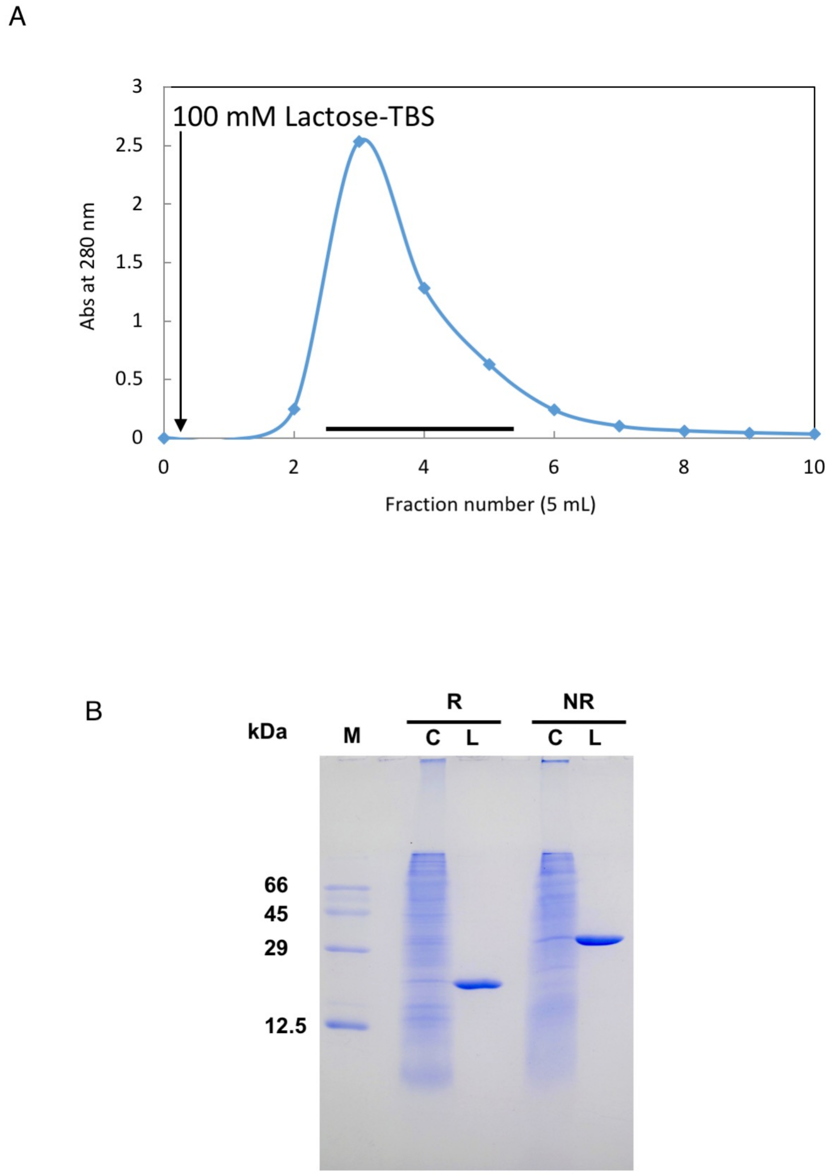

Figure S1. Purification of AJLec. (**A**) TBS-Ca^{2+} extract from the sea anemone *Anthopleura japonica* was applied to a lactose-conjugated cellulose column (1.4 × 3.5 cm) equilibrated with TBS-Ca^{2+}. AJLec that bound to the column was eluted with TBS containing 100 mM lactose (*arrow*). ABS: absorbance. (**B**) an SDS-PAGE pattern under reducing (*R*) and non-reducing (*NR*) conditions. Numbers on the left indicate the molecular weights of marker proteins as follows: BSA (66 kDa), ovalbumin (45 kDa), carbonic anhydrase (29 kDa), and cytochrome C (12.5 kDa). M, molecular marker; C, crude extract; and L, lectin.

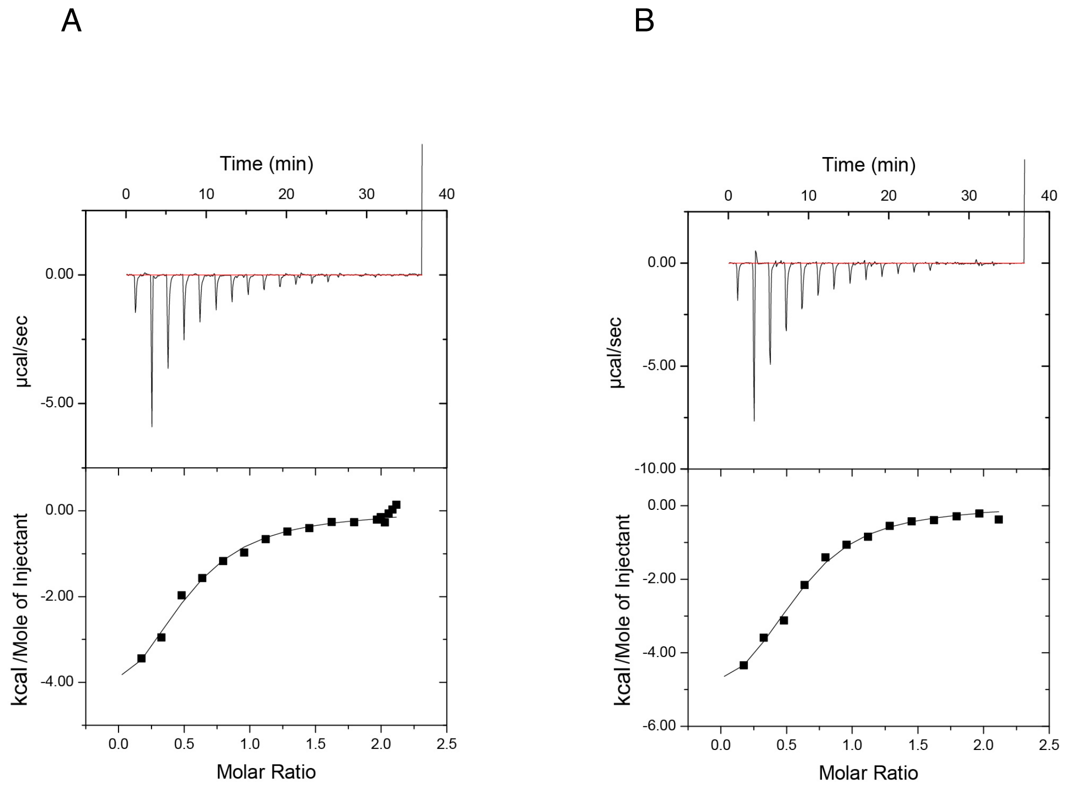

Figure S2. Titration calorimetry results for AJLec with galactose and lactose. (**A**) galactose (5.6 mM) in AJLec (0.56 mM). (**B**) lactose (Galβ1-4Glc) (5.6 mM) in AJLec (0.56 mM). Top: the data from 20 automatic injections of 2 µL of galactose or lactose into a AJLec-containing cell. Bottom: a plot of the total heat released as a function of ligand concentration in the titration shown above (squares). The solid line represents the best least square fit for the resulting data.

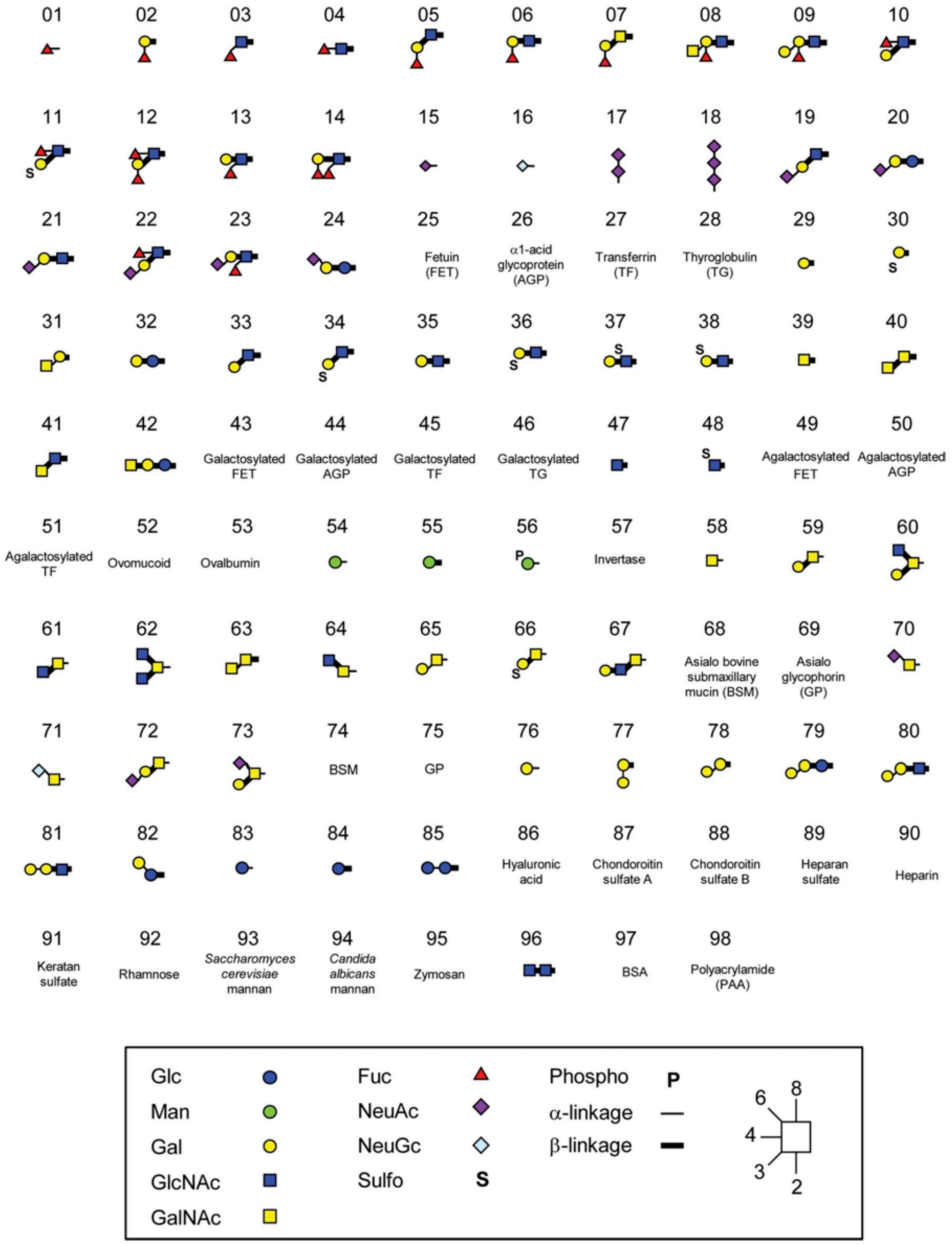

Figure S3. List of oligosaccharides in the glycoconjugate microarray analysis.

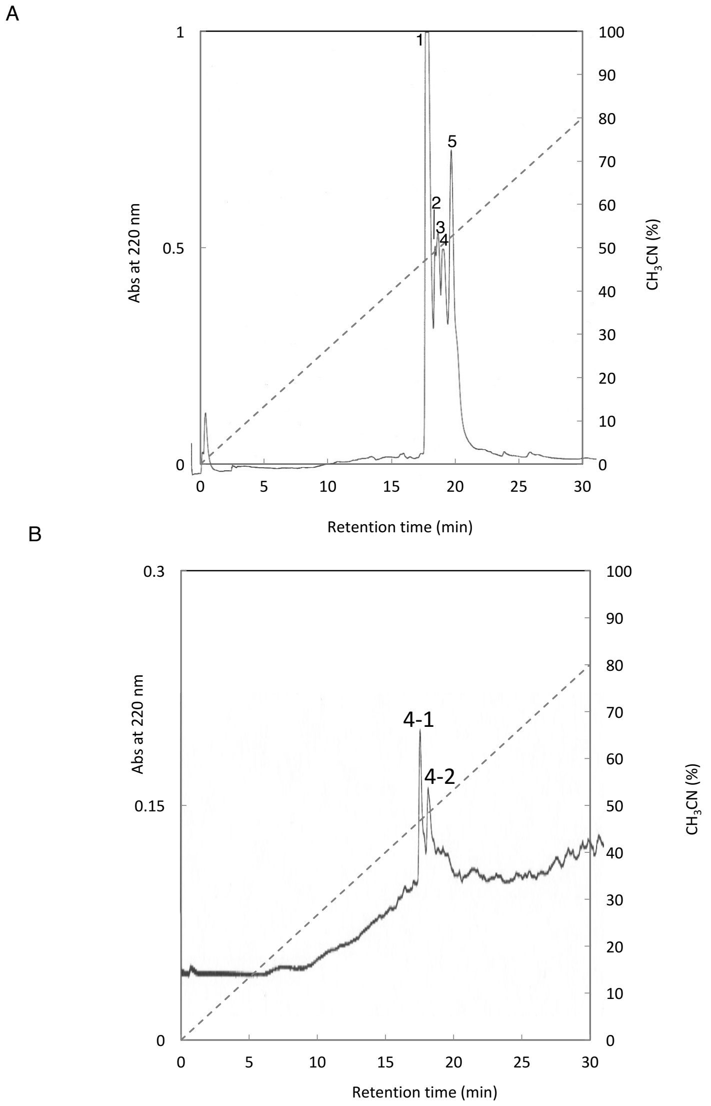

Figure S4. Elution profiles from high-performance liquid chromatography of the peptides. (**A**) Separation of the peptides digested by CNBr cleavage. (**B**) Re-separation from peak fraction number 4 in panel A. The peptides were eluted with a gradient of acetonitrile in dilute aqueous trifluoroacetic acid (TFA).

```
AJLec            -----MKLSTIILVL-----------------------------------VLVGLNLCCS   -3
A7S4R8_NEMVE     --MAKIALLATLLL-------------------------------------VCLLQ----   -4
A7SKF1_NEMVE     -----MKLLLAVL--------------------------------------LCAVVYTEC   -1
A0A2B4RXA2_STYPI -----MSL--ATLLS-----------------------------------MFCLICFLPQ   -5
A0A2B4RXZ1_STYPI MNVHLMSLFAFVFVISRAPKTTSVDIKNDLTPDLSKESRERESLYHVSASHDVPSVKKRS   37
A0A2B4S5E9_STYPI --MSSFSI--KQV---------------------------------------LLVFFCYQ   -5
A0A2B4R6H1_STYPI --MTT-TFFKFVVVV-----------------------------------LISALFIDEA   -3
A0A2B4RWZ9_STYPI ----MKSSFVFLFILN----------------------------------L----SFLAV   -6

AJLec            QGQRCGGWVKLNT-APVCFSAKGNRPGSFTPSH--HGFLKSVKLRHLRGLVTCQSSTDAH   55
A7S4R8_NEMVE     VHAFSGVWIKLNH-GSVCFQAKSNKPGLLLPRH--QGFLAAVKLVHKSGYVSCAGW--SY   52
A7SKF1_NEMVE     HYQTCHEWTKLSE-SRVCFQGQGGKFGKIVPAA--RGFLAAVKLVHREGSIVCGSHPGSH   57
A0A2B4RXA2_STYPI QANGQQIWQQINL-APVCFYASGQKPGEFHYFGD-GRLVAAIKLVYRTGLVRCEKNT-AY   53
A0A2B4RXZ1_STYPI IQSTADRWQKANT-SPVCFGAKHSQFGTFSAP-S-RGLLAAVKLVHLYGYVTCDTRSNRY   94
A0A2B4S5E9_STYPI ILQGEATWHKLNFGLPVCFEARNNRPGYVTYRGSQGILVGALKLVHRSGHVRCVSDV-AY   55
A0A2B4R6H1_STYPI SANVCPRWVKLNK-SPVCFGARSNQYGRFTYPR--NIFVSSFMLVHRSGTVTCNKKN---   52
A0A2B4RWZ9_STYPI KRSAADEWQKQNT-SPVCFGAKNGQFGRFYLKTA-YKKLAAVMLVHLYGYVTCDTRHVSY   53

AJLec            DSYWGCKNRDGFHNYPLNVFVTDKHNKVMFPKTGATYYLDPYVIKNRFYGVQGYNAMSPE  115
A7S4R8_NEMVE     RSHWGCKGL----AYPLNMFVTNAKNQVIFPRVGMKFWPG---NAGRWYSMDGFDSMSND  105
A7SKF1_NEMVE     TSNWGCHNWPHLEKFSINMLITNKDNHVIFPKTGVNFLPK---NPGRWYGMDGFNSRSKE  114
A0A2B4RXA2_STYPI NSRWGCYHHSGYVNYPLNVVITDRHNNILFPLEKFI--KN----AGLWYYLPFTDALHSD  107
A0A2B4RXZ1_STYPI WSFWGCGERSNERP-YVGVVITTSRNHILLPPRRFN--RR----IGKWSKIPGYNSFSSE  147
A0A2B4S5E9_STYPI NSRWGCHGHSSLKSYPLNVVVTDKKNNLIFPLPQFI--KL---SSSLWYEMPVVDDLHSD  110
A0A2B4R6H1_STYPI YSYWGCHPN----NAGLVVVLTDHQNKLLAPAAT---------NSGGWYNLAGYTSSSSA   99
A0A2B4RWZ9_STYPI WSYWGCGSYVHGLG-KINVVITTSDNHVLLPPREFI--VH---NGAKWSKVPGYSSLSPK  107

AJLec            LVLQHGCNSPSDYIGPDSQLRVWYGEDLYNTMESDNSGKVCADVFGYFV-----------  164
A7S4R8_NEMVE     LVLQYGFDQA-YYITPKSVLKVWYGEDLYGYTESDNRGKVCADVYGYFI-----------  153
A7SKF1_NEMVE     LVLTYSFSDP-YFVGPESELRLWYAEDLFNQWEADNRGTVCADVYGYFL-----------  162
A0A2B4RXA2_STYPI ELVFTNYESP-FYLPKGATMKIWYGEDLRKWRNHDNGGRVCVDVYGHLQ-----------  155
A0A2B4RXZ1_STYPI IVLK--SSRP-HSIYKGKGFRVWFGEDLVNYTEGDNAGKVCFDVYILFV-----------  193
A0A2B4S5E9_STYPI ELVFSNFDFP-LYLQPHMELRVWYGEDLKNSHEGDNQGRVCVDVFGYVIGDIEGGHGGSS  169
A0A2B4R6H1_STYPI LVFC-APKKP-HCIFANTELRLWYGEDLRGHTESDNGGRTCADVYGLLA-----------  146
A0A2B4RWZ9_STYPI LMLS--FFSP-SRVSKNQELRVWYGEDLMNAGEGDNGGTICVDVYAIFV-----------  153

AJLec            ---------------------------
A7S4R8_NEMVE     ---------------------------
A7SKF1_NEMVE     ---------------------------
A0A2B4RXA2_STYPI ---------------------------
A0A2B4RXZ1_STYPI ---------------------------
A0A2B4S5E9_STYPI GGRSSQAQALLKDLKESLGNVMQKNWK  196
A0A2B4R6H1_STYPI ---------------------------
A0A2B4RWZ9_STYPI ---------------------------
```

Figure S5. Alignment of the deduced amino acid sequences of AJLec and proteins of *Nematostella vectensis* and *Stylophora pistillata*. The deduced amino acid sequences of AJLec and predicted proteins from *N. vectensis* (starlet sea anemone) and *S. pistillata* (Smooth cauliflower coral). AJLec and predicted proteins (UniProt codes: A7S4R8_NEMVE, A7SKF1_NEMVE, A0A2B4RXA2_STYPI , A0A2B4RXZ1_STYPI, A0A2B4S5E9_STYPI, A0A2B4R6H1_STYPI, and A0A2B4RWZ9_STYPI) were aligned in Clustal Omega software. Residues identical in AJLec and the predicted proteins are marked in red. Residues in the signal sequence of the proteins are colored light blue. Residues interacting with carbohydrates, the Ca^{2+} ion in the sugar-binding site, and the second Ca^{2+} ion are denoted by red, blue, and green asterisks, respectively. α-Helices and β-strands in AJLec are indicated as boxes in orange and yellow, respectively.

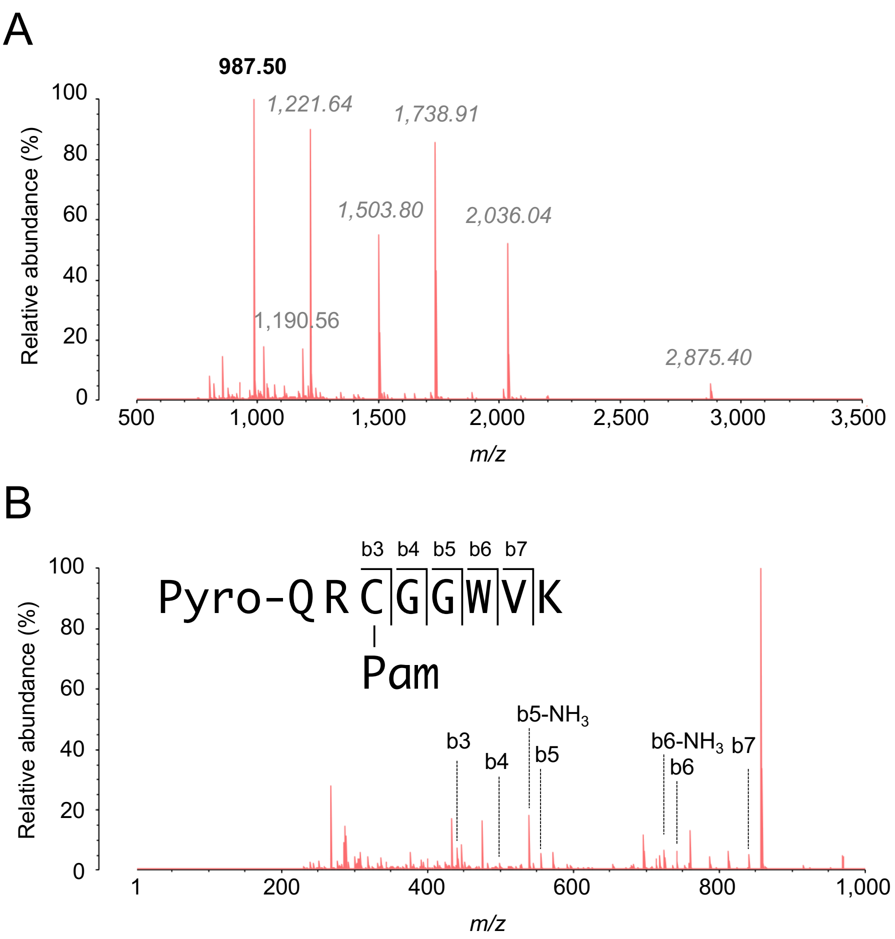

Figure S6. Mass spectrometric analysis of the N-terminal peptide from the lysyl endopeptidase digest of Pam-AJLec. (**A**) MS spectrum showing five internal peptides (shown in gray italics: m/z 1,221.64, positions 9–19, LNTAPV[Pam-C]FSAKLNTAPV[Pam-C]FSAK; m/z 1,503.80, positions 88–100, TGATYYLDPYVIKTGATYYLDPYVIK; m/z 1,738.91, positions 20–35, GNRPGSFTPSHHGFLKGNRPGSFTPSHHGFLK; m/z 2,036.04, positions 63–79, NRDGFHNYPLNVFVTDKNRDGFHNYPLNVFVTDK, and m/z 2,875.40, positions 39–62, LRHLRGLVT[Pam-C]QSSTDAHDSYWG[Pam-C]K) and the C-terminal peptide (shown in gray: m/z 1190.56, positions 155–164, V[Pam-C]ADVFGYFV) that were judged by PMF (protein score 66). The most prominent ion (m/z 987.50, shown in bold) was not assigned by PMF (see text). (**B**) Annotated MS/MS spectrum of the N-terminal peptide (m/z 987.50 as a precursor).

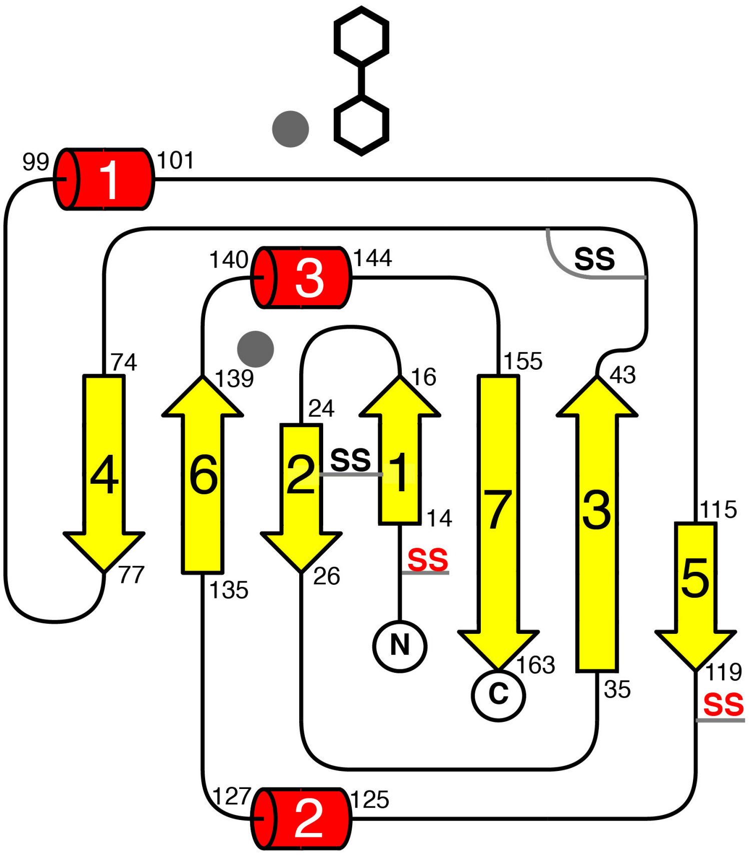

Figure S7. Topological diagram illustrating the secondary structural elements of AJLec. Arrows and cylinders represent β-strands and α-helices, respectively. Stick model and gray circles are shown as lactose and Ca^{2+} ions, respectively. Residue numbers at first and end of the secondary structures are shown. Intra- and inter-molecular disulfide bridges are shown as black and red symbols of SS, respectively.

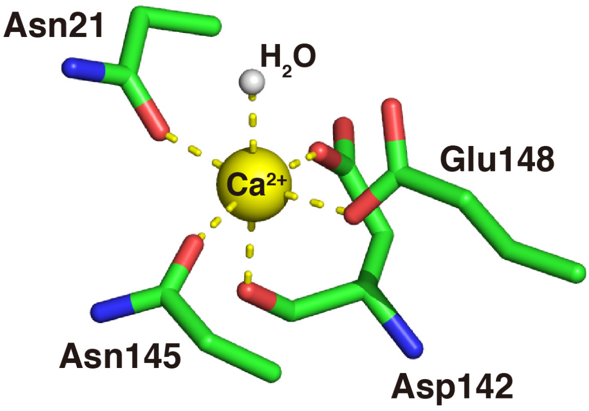

Figure S8. Second binding-site for Ca^{2+} ion in AJLec. Residues coordinating to the Ca^{2+} ion are shown as stick figures. Ca^{2+} ion and water molecule are shown as yellow and gray spheres, respectively. coordinating bonds are shown as dotted lines.

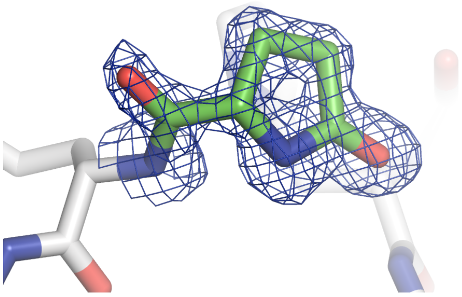

Figure S9. N-terminal modification of AJLec. The modification of the pyroglutamic acid on the N-terminal residue in mature AJLec is shown as a green stick figure. The $2F_o$–F_c electron density map is shown as a blue mesh. The contour level of the map is 1.0σ.

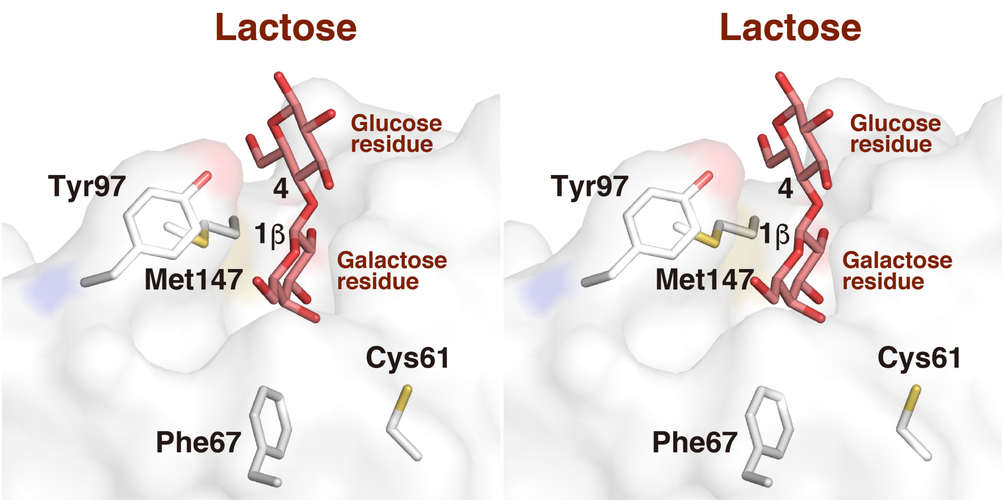

Figure S10. Stereo Figure of lactose and adjacent residues in AJLec. Lactose, Met147, Tyr97, Phe67, and Cys61 are shown as stick figures. Protein surface is shown as translucent description.
